# Evaluation of a software system for estimating planned dose error in patients, based on planar IMRT QA measurements

**DOI:** 10.2478/raon-2013-0042

**Published:** 2014-01-22

**Authors:** Mohammad Bakhtiari, Ashkan Parniani, Fritz Lerma, Shannon Reynolds, James Jordan, Alireza Sedaghat, Mehrdad Sarfaraz, James Rodgers

**Keywords:** intensity modulated radiation therapy, quality assurance, 3D dosimetry, planar dosimetry, MapCheck, 3 dose-volume histogram

## Abstract

**Background:**

Intensity modulated radiation therapy (IMRT) dosimetry verification is routinely conducted *via* integrated or individual field dosimetry using film or a matrix of detectors. Techniques and software systems are commercially available which use individual field dosimetry measurements as input into algorithms that estimate 3D patient dose distributions on CT scan derived target volumes and organs at risk (OARs), thus allowing direct dose-volume histogram (DVH) analysis *vs*. treatment planning system (TPS) DVH. The purpose of this work is to present a systematic benchmarking technique to evaluate the accuracy and consistency of such a software system.

**Methods:**

A MapCheck2 diode array and 3DVH™ software from Sun Nuclear were used for this study. Delivered planar dose was measured with the diode array as an input to 3DVH™ software that was used to estimate the 3D dose matrix. Accuracy of the output of 3DVH™ is tested by comparing measured planar doses over a range of depths to the same planes reconstructed by 3DVH™. Different fields from complex IMRT cases were selected and examined in this study. The sensitivity to depth of measurement was evaluated.

**Results:**

The Gamma Index analysis, comparing calculated 3D dose with measured 3D dose with 2% and 2mm distance-to-agreement (DTA) criteria returned a pass rate of > 90% for all patient cases calculated by the treatment planning system and it returned a pass rate of > 96% in 9 out of 10 cases calculated by 3DVH™. Extracted computed dose planes with 3DVH™ software at different depths in the flat phantom passed all gamma evaluation analyses when compared to measured planes at different depths using MapCheck2.

**Conclusions:**

Studying complex head and neck IMRT fields, it was shown that the 3D dose distribution predicted by the planned dose perturbation (PDP) algorithm is both accurate and consistent.

## Introduction

The level of complexity and uniqueness of intensity modulated radiation therapy (IMRT) for each patient requires accurate and precise dosimetry verification and quality assurance (QA) technique(s).^1^,^2^ QA usually can be carried out in the form of either composite or individual field dosimetry.^3^ With the composite dosimetry approach, the composite dose distribution is measured in one or more selected phantom planes. For individual field dosimetry a flat phantom is used to measure the dose distribution in a plane perpendicular to the beam axis of each individual field.^3^ The individual beam approach leads to useful information about the sources of discrepancy in the planning and delivery process of each individual field. The measurements could be done either using film or a matrix of detectors.^4^–^11^ There are other techniques available that do not use a flat phantom.^12^ Recently it was demonstrated that the individual beam approach may not be adequate in investigating the accuracy of dose delivery to individual organs at risk.^13^,^14^ The conventional IMRT QA is performed for a plane normal to the beam axis using passing rates for Gamma index^15^ or %/DTA composite;^16^ as such, they do not provide much information about impact in 3D, *i.e*. the actual deliverable dose volume histograms of different organs. Therefore, obtaining a 3D dose distribution is desirable for an improvement in the sensitivity and specificity of IMRT QA analyses. A 3D dose distribution can be estimated in several ways such as: 1) measuring dose in a volume in a 3D dosimeter or 2) using a software system that estimates the 3D dose using phantom measurements and phantom calculations as input to guide a reconstruction algorithm.^17^ Method 2 provides a volumetric planar dose distribution by modifying planned dose or reconstructing dose using measured planar dose and therefore we name it as a *virtual measurement* of the 3D dose distribution. This method may be useful if the accuracy of the algorithm is verified.

Currently there are several commercially available planar software systems that estimate 3D patient dose deviations based on inputs from 2D measurements such as the COMPASS system (IBA-Wellhofer), DOSIMETRYCHECK (Math Resolutions LLC), and 3 dose-volume histogram (DVH) (Sun Nuclear Corporation). Systematic benchmarking techniques must be developed in order to verify the accuracy of the algorithms behind them. Others have published studies on the accuracy of the planned dose perturbation algorithm (PDP) using planar measurement^18^ and simulation.^17^ In this investigation we developed another evaluation technique using measurements over a range of depths. The measured 2D dose matrices were used to compare to virtual measurements to verify the accuracy of the algorithm. Although the software system 3DVH™ is benchmarked here, the technique can be used for benchmarking other similar software systems as well.

## Methods

In a systematic way, dose matrices were obtained in three different ways for inter-comparison: I) Using the treatment planning system in a homogeneous water phantom (3D dose), II) *via* multiple planar measurements over a range of depths, and III) *via* the 3DVH™ perturbation algorithm based on measurements and calculations from a single planar depth (3D dose). For methods II and III, a 2D diode array (MapCheck2 from Sun Nuclear, Melbourne, FL) was used for measuring the individual fields. For the PDP algorithm, two main components are used: 1) the 3D dose calculation exported from the treatment planning system (TPS) as the unperturbed planned dose *D(x,y,z)* and 2) a modeling that perturbs the planned dose component *d(x,y,z)* using QA phantom measurements vs. QA TPS calculations. In order to obtain a 3D perturbed dose matrix both the planar measurements at a certain depth and TPS calculated planar dose at the same depth are needed. If there is any dose differences found between the MapCheck measurement and the TPS dose calculation for each beam, the software uses those dose differences and projects them back into the TPS 3D dose calculation to obtain an estimate of the actual delivered 3D dose distribution.^18^ A recent investigation verified accuracy of the PDP algorithm by introducing known errors and comparing the known effects versus the predicted effects.^17^ In this work, we use similar strategy but use actual measurements at different depths to verify the 3DVH estimations at those depths.

**FIGURE 1.**
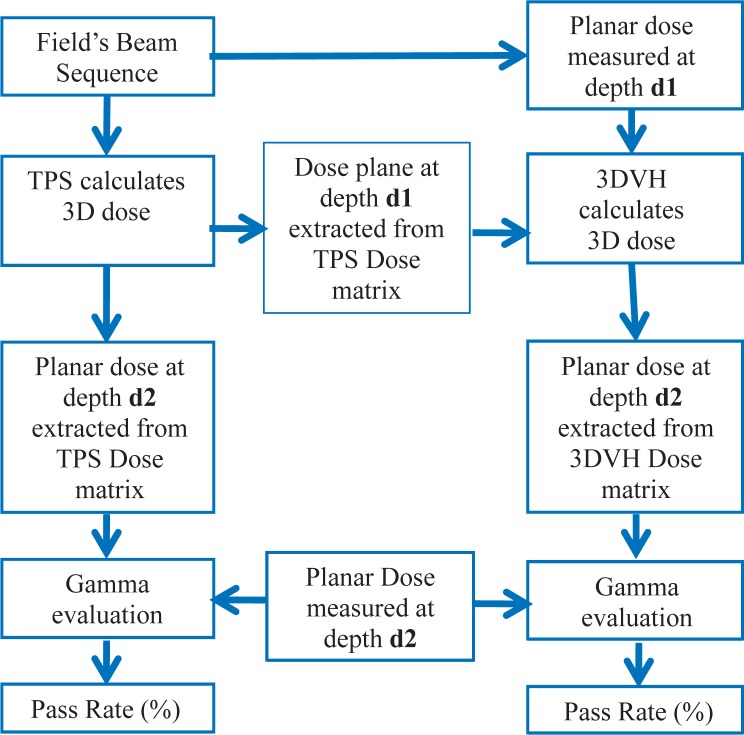
The flow chart of the benchmarking technique. Both treatment planning system (TPS) and 3DVH^™^ return a 3D dose matrix. The 3DVH^™^ dose matrix is reconstructed using the measurements and TPS data on a plane at depth d1. Additional measurement at a different depth of d2 is carried out. The new measurement at depth d2 was compared to the corresponding planar dose extracted from the calculated 3D doses in TPS and 3DVH^™^.

**FIGURE 2.**
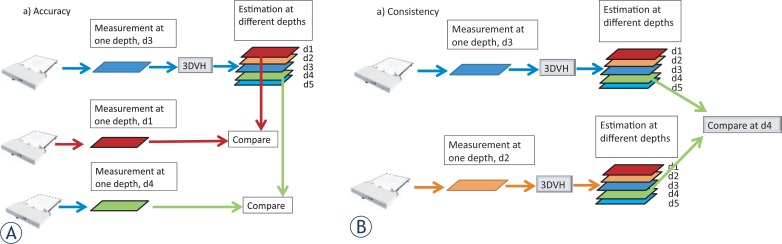
Schematic diagram of the experimental setup to benchmark 3 dose-volume histogram (DVH).

The flow chart of the benchmarking technique is shown in Figure 1. The beam’s multileaf collimator (MLC) segments for each field are used in the TPS to calculate a 3D dose matrix in a cubic water phantom for each field individually. The planar dose at depth d1 and d2 are extracted from the 3D dose matrix calculated by the TPS. The same programmed MLC segments are used to deliver and measure the planar dose at a depths d1 and d2 using MapCheck with rectangular solid water equivalent build up. The measured and TPS calculated planar doses at depth d1 are compared to calculate a 3D dose matrix using 3DVH™ software (Figure 2). Then, the planar dose at a different depth (such as d2, d3, or d4) was extracted from the 3D dose calculated by 3DVH™. For the measurements and calculations we kept the SSD fixed at 95 cm. Figure 2 also shows a schematic diagram of the experimental setup to benchmark 3DVH.

The MapCheck device calibration requires a relative array calibration and an absolute calibration. The array calibration is usually independent of the depth and can be done annually.^4^ The absolute calibration at each depth is recommended to be done every time the MapCheck is set up for measurement.^19^ We did the array calibration at depth 2 cm as recommended by manufacture and we did the absolute calibration for each depth separately.

Complex IMRT plans (three head and neck cases and one complex prostate case) were selected for the study. About twenty fields were randomly selected for checking the accuracy. For each field the 3D dose distributions were calculated in the TPS. Analytical anisotropic algorithm (AAA) dose computation algorithm was used in the Eclipse TPS (Varian Medical Systems, Palo Alto, CA).

### Accuracy

The 3DVH^™^ dose matrices were calculated using the planar TPS and measurements at depth d1 = 5 cm. Comparisons of calculated planar dose matrices (from TPS and 3DVH^™^) were evaluated at depths d2 = 7 and 9 cm (Figure 2A). The gamma evaluation software returns the pass rate (%) using a distance to agreement of 2 mm and dose difference 2%.

### Consistency

The 3DVH^™^ dose matrix calculated by measured data should be independent of the depth of measurements, that is the 3DVH^™^ dose distribution obtained with measurements at depth d1 = 7 cm should be almost identical to those obtained with measurements at depth d1 = 9 cm. The 3DVH^™^ code was run with measurements at depths d1 = 7 and 9 cm and the extracted planar doses were compared with measurements at depths d2 = 5 cm (Figure 2B).

An additional investigation of the consistency of the 3DVH algorithm was assessed by interrupting one of the IMRT fields half way through its delivery. Therefore half of the monitor units (MUs) were delivered (We simply pushed the beam off button when half of the beam MU was delivered). Using 3DVH^™^ the composite 3D dose and DVHs were calculated using beam measurements at different depths.

In this paper we have concentrated on field by field measurements. Recently there was also an investigation on the 3DVH accuracy based on cumulative/composite measurements.^18^ In Ref.^18^ the results were benchmarked with film and ion chamber. We also, as the last benchmark, did the same investigation but with MapCheck as the only measurement tool to finalize our study. A head and neck patient’s fields were measured at a depth in 2 different ways: (I) measuring the individual fields at depth 5 cm with gantry angle set to zero and using these data in 3DVH^™^ to obtain a 3D dose distribution, and (II) Measuring a composite planar dose distribution at depth 5 using MapPhan and actual gantry angle of each beam.

## Results

### Accuracy

**FIGURE 3.**
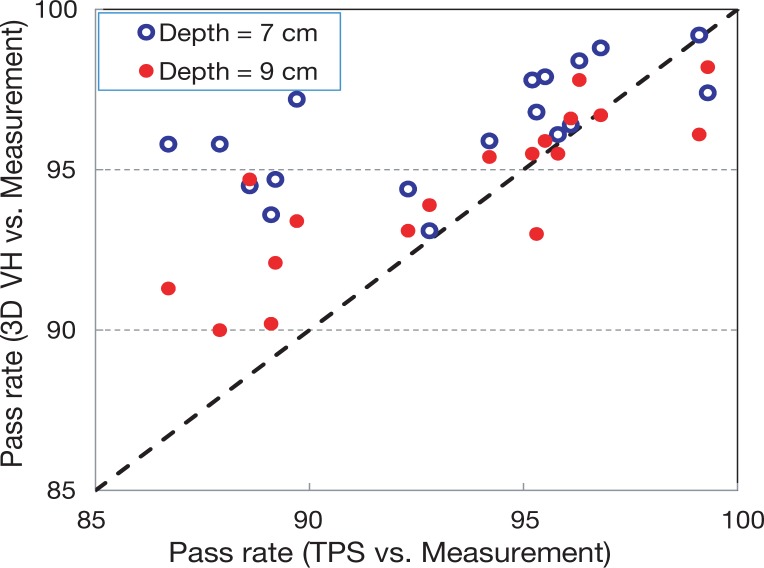
3D doses were obtained using measurements and treatment planning system (TPS) planar doses at d1 =5 cm. The planar doses extracted at depths d2 = 7 and 9 cm from the calculated 3D doses are compared to measurements at corresponding depths. The gamma evaluation software returns the pass rate (%). The 3DVH^™^ estimates of dose are closer to the direct measurement than the original TPS calculation, as indicated by the points above the dotted line.

In Figure 3, the pass rates calculated by comparing the measurements versus planar doses from the TPS and 3DVH^™^ are shown. In Figure 3, it is shown that there is a correlation between the pass rate of 3DVH^™^ dose planes and those from the TPS when the pass rate is high. Pass rates of greater than 90% are seen for the 3DVH^™^ data. On the other hand, comparing the measurements and TPS calculations leads to pass rates greater 86%. Most importantly, the 3DVH^™^ agreement *vs*. measurement is better than the TPS agreement as indicated by the vast majority (9 out of 10) of points residing above the dotted line on Figure 3; this is the goal of the perturbation software, that is, to be an improvement on the TPS prediction. The reason could be the MLC characterization in TPS that leads to differences between planned and delivered dose.

**FIGURE 4.**
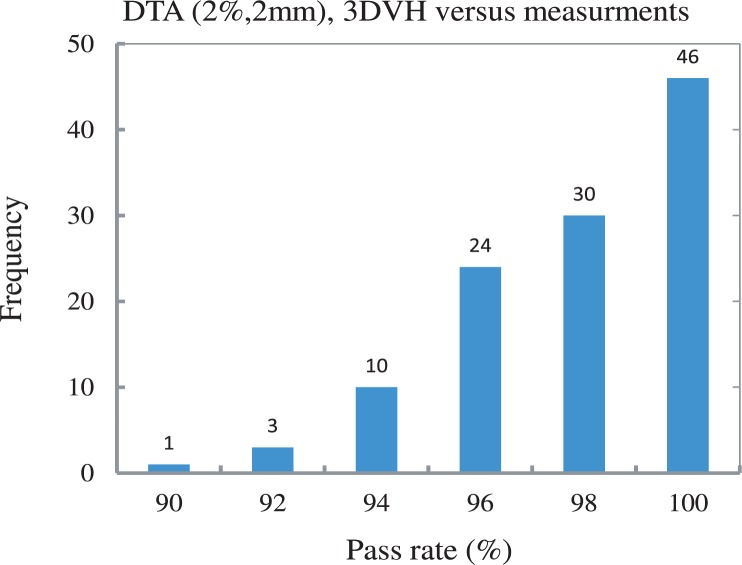
Bar chart of the pass rates obtained by comparing 3DVH^™^ outputs with measurements.

**FIGURE 5.**
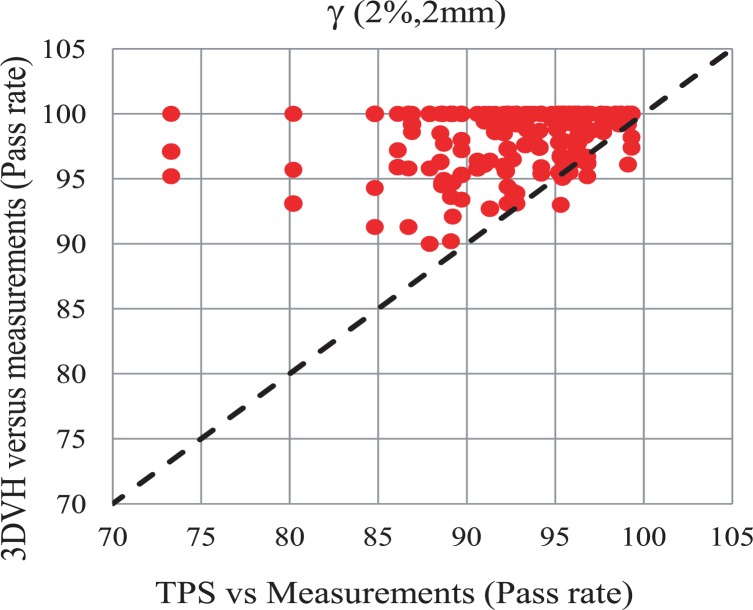
Comparison between treatment planning system (TPS) and DVH^™^ pass rate at 3 different depths. Again, the accuracy vs. measurement of 3DVH^™^ was an improvement over the original TPS dose.

Similarly the measurement at d1 = 7 cm or d1 = 9 cm were used to run 3DVH^™^ and the results were compared with the measurements in other depths. A bar chart of all of the comparisons between 3DVH^™^ outputs and the measurements are shown in Figure 4. Approximately 90% of the data points have a pass rate of more than 96%. The comparison of these data with TPS is shown in Figure 5. Interestingly, when comparing to the TPS, the 3DVH^™^ results show a better agreement with measurements. This might be expected since 3DVH^™^ is calculated based on the delivered dose not calculated.

### Consistency

**FIGURE 6.**
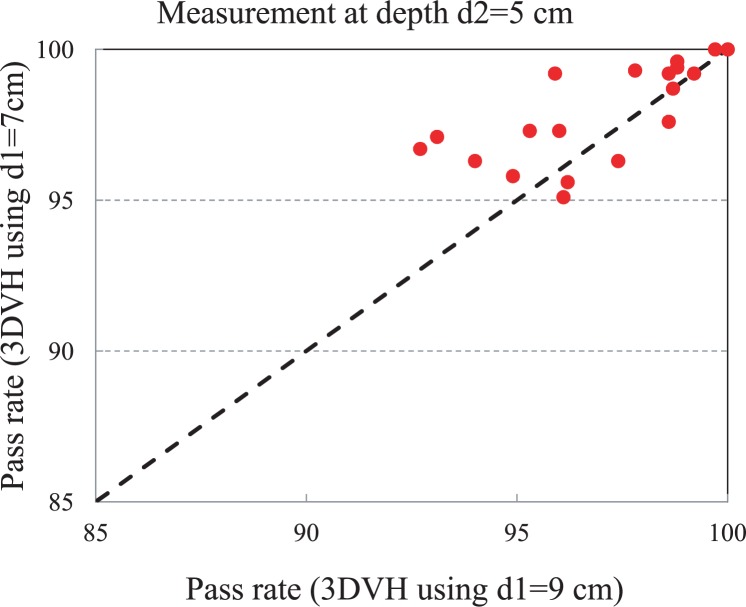
Two 3DVH^™^ dose matrices calculated using treatment planning system (TPS) and measured planar data at d1 = 7 and 9 cm. The extracted planar dose at d1 = 5 cm is compared with measurement. There is a correlation between both dose matrices.

**FIGURE 7.**
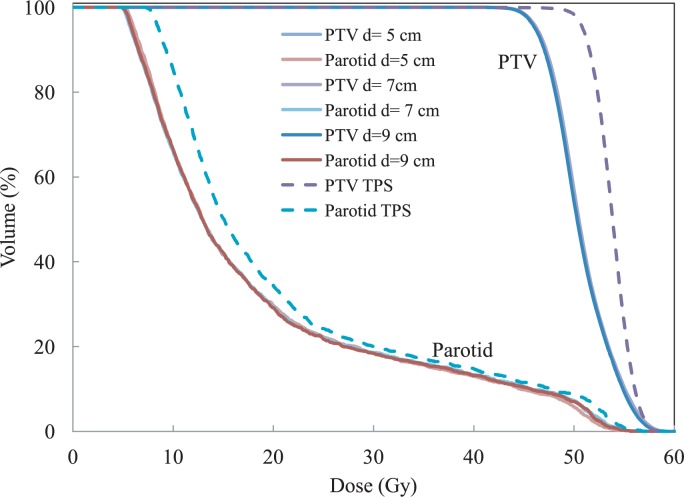
The dose-volume histogram (DVH) curves for different organs planned by the treatment planning system (TPS) (dashed lines) and calculated by 3DVH^™^ (solid lines). The input to 3DVH^™^ was measurements done by introducing a delivery error by interrupting the one of the beam’s delivery half way through. The DVH curves from 3 different 3DVH^™^ files obtained at depths 5, 7, and 9 cm, three solid lines, are overlapped, indicating the consistency of the algorithm in detecting error.

The 3DVH^™^ planar dose code was run with measurements at depths d1 =7 and 9 cm and the extracted planar doses were compared with measurements at depths d2 = 5 cm (Figure 2B). The results are shown in Figure 6. There is a correlation between the data sets, however it seems there is a trend toward the depth dependence. In order to investigate this more, the beam delivery of one of the fields was interrupted half way through and all of the IMRT fields were measured at 3 different depths. The resulting DVH curves are shown in Figure 7. The DVH curves obtained from 3DVH^™^ at different depths overlap each other, showing a clear error in the delivery, as expected.

**FIGURE 8.**
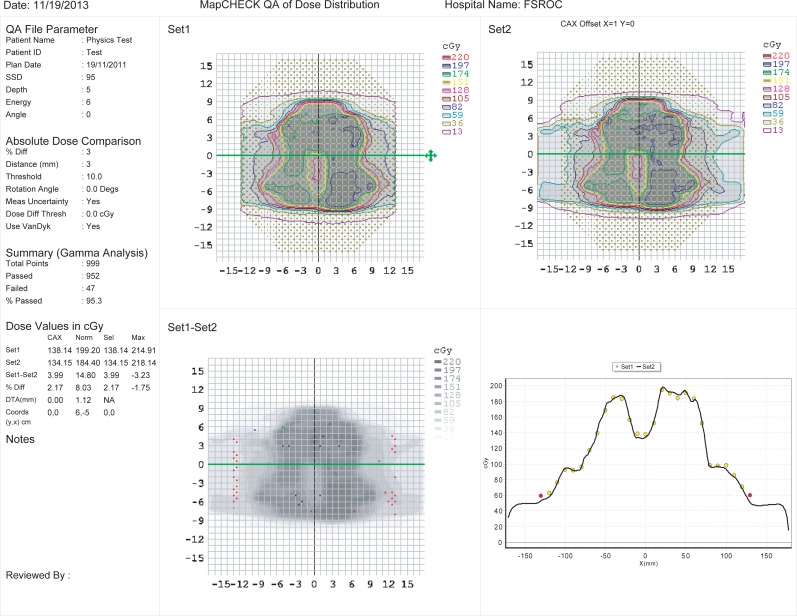
The composite measurement of a 9-field head and neck treatment planning system (TPS) case (set 1) is compared to the dose distribution obtained from 3DVH^™^ (set 2). The 3DVH^™^ software was run using individual field’s measurement at fixed gantry angle of zero. The composite measurement was done by adding up individual field’s measurement at their actual gantry angle. The resulted pass rate is 95.7%.

The data from a composite calculation by 3DVH are compared with direct composite measurement by MapCheck in Figure 8. The gamma evaluation pass rate with 3 mm, 3% is about 96%. It is noted that MapCHECK/MapPhan has known inaccuracy for lateral beams due to measurement errors at these angles.^4^ 3DVH^™^ allows 3D composite dosimetry that is not subject to these errors and which estimates a full volume rather than a single plane of dose.

## Discussion

In Figure 3 it was shown that the dose matrices estimated by 3DVH are closer to direct measurements compare to the dose matrices calculated by TPS. The differences between delivered and calculated dose may come from MLC characterization in TPS (leading to calculation errors) or failure of MLCs in delivering the planned motions accurately.

In order to take out the effect of the consistency of MLC segment delivery on the results, we carried out the measurements in different days. Although MLC segment delivery errors are constrained by Linac manufacturer interlocks such that their magnitude cannot be large, it is also true, that small errors can add up to a large dosimetric error. For this study, we are focusing on systematic errors, and patient-specific errors (such as over-modulation leading to a very poor deliverability) that can be caught from just one fractional delivery.

The analysis done in Ref.^20^ has shown that the spaces between diodes in comparison to films do not play a significant role in determining the final results of QA procedure.^20^ The 3DVH software uses “Smarterpolation” as the interpolation technique, which means that it shapes the lines in between measurement point using the TPS plan’s shape. We do not have any statistics or systematic study whether this may cause significant effect on the results. This should be addressed in future studies.

Regarding the inhomogeneity corrections, because 3DVH begins its calculation of patient dose from the starting point of the treatment planning system dose and only perturbs the dose based on measured errors, the dose already has the inhomogeneity information. The TPS has calculated the dose based on the heterogeneities in the CT scan.

It is seen in Figures 3 and 5 that some of the data points are below the diagonal line indicating lower pass rate with 3DVH in comparison to TPS. This research was designed to benchmark the 3DVH code on a single field basis. The 3DVH has been basically developed to obtain a composite dose error matrix from several single field measurements. An error in the setup or measurement can easily penetrate into other depths in single field analysis. Nevertheless, further investigation is needed for such cases.

## Conclusions

A method was developed to investigate the accuracy and consistency of the software systems that approximate the dose distribution in the patients based on planar measurements. The planar measurements can be carried out by film exposures or diode matrices (such as MapCheck). One of such software systems (3DVH^™^ from SunNuclear) was examined and it was found that:

The software results are independent of the depth of planar measurement, indicating the consistency of the results.

The 3D dose distributions obtained by 3DVH^™^ were closer to measurements compare to those from TPS, suggesting the accuracy of the software and usefulness of the software for *estimating* dose differences at other depths.
